# Higher mortality of the less suitable brown trout host compared to the principal Atlantic salmon host when infested with freshwater pearl mussel (*Margaritifera margaritifera*) glochidia

**DOI:** 10.1007/s00436-021-07145-4

**Published:** 2021-04-12

**Authors:** Janhavi Marwaha, Per Johan Jakobsen, Sten Karlsson, Bjørn Mejdell Larsen, Sebastian Wacker

**Affiliations:** 1 Department of Biology, University of Bergen, Thormøhlens Gate 53A, 5006 Bergen, Norway; 2 Norwegian Institute for Nature Research (NINA), Postboks 5685 Torgarden, 7485 Trondheim, Norway

**Keywords:** *Margaritifera*, Glochidia, Host-parasite, Virulence, Mortality, Host bias

## Abstract

The freshwater pearl mussel (*Margaritifera margaritifera*) is a highly host-specific parasite, with an obligate parasitic stage on salmonid fish. Atlantic salmon (*Salmo salar*) and brown trout (*Salmo trutta f. trutta* and *Salmo trutta f. fario*) are the only hosts in their European distribution. Some *M. margaritifera* populations exclusively infest either Atlantic salmon or brown trout, while others infest both hosts with one salmonid species typically being the principal host and the other a less suitable host. Glochidial abundance, prevalence and growth are often used as parameters to measure host suitability, with the most suitable host species displaying the highest parameters. However, it is not known if the degree of host specialisation will negatively influence host fitness (virulence) among different host species. In this study we examined the hypothesis that glochidial infestation would result in differential virulence in two salmonid host species and that lower virulence would be observed on the most suitable host. Atlantic salmon and brown trout were infested with glochidia from two *M. margaritifera* populations that use Atlantic salmon as their principal host, and the difference in host mortality among infested and control (sham infested) fish was examined. Higher mortality was observed in infested brown trout (the less suitable host) groups, compared to the other test groups. Genetic assignment was used to identify offspring from individual mother mussels. We found that glochidia from individual mothers can infest both the salmonid hosts; however, some mothers displayed a bias towards either salmon or trout. We believe that the differences in host-dependent virulence and the host bias displayed by individual mothers were a result of genotype × genotype interactions between the glochidia and their hosts, indicating that there is an underlying genetic component for this parasite-host interaction.

## Introduction

Parasites are typically classified as either being host specialists or host generalists depending on their host range; i.e. the former attain high fitness on very few host species compared to the latter, which attain it on many (Veiga et al. 1998; Poulin 2007; Leggett et al. 2013; Lievens et al. 2018). Host specificity is both a reflection of the individual biological properties of the parasite and the host, as well as a result of the specific interaction between the two (Dick and Patterson 2007). Moreover, the degree of host specificity displayed by a parasite is believed to be a result of the historical associations between the parasite and its hosts and present ecological events (Dick and Patterson 2007; Poulin 2007). It is an important attribute of a parasite because it has an influence on its ecology and future evolution; for example, the ability of the parasite to adapt to new hosts (Poulin and Mouillot 2003; Salvaudon et al. 2007; Lievens et al. 2018). In order to understand how the degree of host specificity will influence parasitic fitness, and their survival in a changing environment, it is important to measure their fitness across different host species (Lievens et al. 2018).

Most studies on host-parasite relationships involve short-lived parasites. Host-parasite interactions involving a long-lived parasite, and the effect of these interactions on parasite fitness, are not well understood. Parasites are usually regarded as having a greater evolutionary potential and adaptive plasticity, resulting from them having larger population sizes, higher mutation rates and shorter generation times compared to their hosts (Ebert 1994; Kaltz and Shykoff 1998; Gandon and Michalakis 2002; Dybdahl and Storfer 2003). The freshwater pearl mussel (FPM), *Margaritifera margaritifera*, is an example of a long-lived specialist parasite with a reproductive lifespan that is 30 times longer than its host (Geist and Kuehn 2008), and it is a good model to examine the influence of high host specificity on parasite fitness, in particular virulence and infectivity (host bias).

*M. margaritifera* is a freshwater bivalve which belongs to the order Unionida, and like all unionid mussel species, it has a complex life cycle. The FPM life cycle includes an obligate parasitic stage on a suitable salmonid host (Smith 1976; Meyers and Milleman 1977; Young and Williams 1984a). Parasitic glochidia (60–80 μm) released by gravid mothers reach a fish host passively by drifting with the water current and are encysted by gill epithelial cells of a suitable host (Young and Williams 1984a; Bauer 1987). Infective glochidia are not selective during attachment and are able to attach to all objects (e.g. wood, plastic or paper) (Kat 1984; Dodd et al. 2005), but in order to be encysted by gill epithelial cells of the host, they must induce an immune response (Nezlin et al. 1994; Jansen et al. 2001). Glochidia that are unable to induce an immune response are shed off (Nezlin et al. 1994). Host-parasite compatibility is believed to be an underlying factor that influences successful glochidial encystment on suitable hosts, duration of the parasitic phase and post-parasitic performance of juvenile mussels (Haag 2012; Marwaha et al. 2017; Taeubert and Geist 2017). After a parasitic period lasting between 9 and 11 months, free-living juvenile mussels excyst and spend the next 5 years buried in the river substratum (Smith 1976; Bauer 1987).

Unionid mussel species display varying degrees of host specificity. This can range from host generalists, such as *Anodonta* species that metamorphose on several host species, to highly host-specific ones, such as those of the family Margaritiferidae that can develop only on a few closely related host species (Bauer 2001; Strayer et al. 2004). *M. margaritifera* displays a high degree of specialisation, and Atlantic salmon (*Salmo salar*) and brown trout (*Salmo trutta f. fario* and *Salmo trutta f. trutta*) are their only hosts in their European distribution (Young and Williams 1984b). Some FPM populations can exclusively infest either Atlantic salmon (‘salmon-mussels’) or brown trout (‘trout-mussels’) even when both host species are present (Larsen et al. 2000; Hastie and Young 2001; Karlsson et al. 2014; Österling and Wengström 2015; Salonen et al. 2017; Wacker et al. 2019); whereas others infest both salmonid host species but with varying degrees of suitability (Taeubert et al. 2010; Salonen et al. 2017; Taeubert and Geist 2017; Clements et al. 2018). Highly host-specific FPM populations have been observed in rivers in Ireland (Geist et al. 2018), Scotland (Hastie and Young 2001), Sweden (Österling and Wengström 2015) and Norway (Larsen et al. 2012; Karlsson et al. 2014; Wacker et al. 2019). High host specificity has also been observed in artificial infestation experiments, in which salmon- or trout-mussels, exposed to both salmonid host species in the same infestation tank, only infested the principal salmonid host (Larsen et al. 2012; Österling and Wengström 2015; Wacker et al. 2019). In FPM populations that infest both salmonid hosts (i.e. a principal and a less suitable host), it is not clear if glochidia from specific families exclusively infest either brown trout or Atlantic salmon or if offspring from the same family can infest both the salmonid hosts. Host suitability studies, which determine the salmonid species requirements of a particular FPM population, provide the essential information required for conservation efforts such as artificial breeding programmes and re-stocking of suitable hosts in FPM rivers (Salonen et al. 2017; Taeubert and Geist 2017; Clements et al. 2018).

Glochidia take 9–11 months to metamorphose, and glochidial survival depends on host survival for this entire duration. In the FPM host-parasite relationship, the parasite is expected to experience a stronger selection pressure on compatible host genotypes because its survival depends on host compatibility (Douda et al. 2017). In comparison, the hosts are expected to experience a weaker selection for resistance to glochidia (Douda et al. 2017). This is because the parasite is distributed across a smaller area of the host’s total distribution range, and it infests only the freshwater (young) stage of the host (Douda et al. 2017). However, glochidial infestation has a negative effect on the host and causes an increase in blood haematocrit values, spleen enlargement, respiratory stress and impaired swimming (Taeubert and Geist 2013; Horký et al. 2014; Thomas et al. 2014; Douda et al. 2017; Filipsson et al. 2017; Marwaha et al. 2019). Typically, low to moderate glochidial infestation has no significant detrimental effect on hosts, while high glochidial loads can lead to host mortality (Treasurer et al. 2006; Taeubert and Geist 2013). However, previous studies have shown that glochidial densities which were within the recommended range on a host fish (5–100 per gram fish) (Taeubert and Geist 2013) resulted in respiratory stress (Thomas et al. 2014; Marwaha et al. 2019). Thus, the resulting cost of infestation to the host suggests that pearl mussel glochidia are indeed a selective force, and this can result in potential mussel-salmonid host coevolution (Douda et al., 2017; Chowdhury et al. 2019).

Virulence is defined as the reduction in host fitness (mortality) as a result of parasitic infestation (Bull 1994; Read 1994; Dybdahl and Storfer 2003; Lambrechts et al. 2006; Bieger and Ebert 2009). Parasitic virulence is not a trait of the parasite alone but is believed to be a result of either a parasite’s adaptive strategy, host response to parasite infestation, or a complex interaction between these two (Ewald 1983; Mackinnon et al. 2002, Day and Burns 2003; Perlman and Jaenike 2003). Some studies have shown that a parasite can cause differential virulence or mortality among closely related host species (Thomas et al. 1995; Hurst and Bartholomew 2012; Lievens et al. 2018). Lievens et al. (2018) observed differential virulence of a microsporidian parasite (*Anostracospora rigaudi*) among two species of brine shrimp (*Artemia parthenogenetica* and *Artemia franciscana*). This was dependent on the degree of compatibility between the parasite and the host. Differences in host species (and strain) susceptibility to different FPM populations are well documented. To the best of our knowledge, host species–dependent differences in glochidial virulence (mortality) in FPM have not been examined.

The purpose of this study was to investigate if there was a difference in mortality between brown trout and Atlantic salmon that have been infested with salmon-mussel glochidia. We hypothesised that glochidial virulence, measured as host mortality, would be higher on the less suitable brown trout host. We also examined variation in the infestation success of offspring from individual mother mussels on the two salmonid hosts. We hypothesised that individual FPM mothers can infest both host species, but with varying degrees of success. In order to test our hypotheses, we used glochidia from two FPM populations that have been observed to use Atlantic salmon as the principal host, to infest both Atlantic salmon and brown trout (Johnsen et al. 2008; Eilertsen et al. 2018). We used hatchery-reared (naïve) fish of the same age in our experiment. This might be expected to limit the possible effects from local genetic adaptation in the host. However, we cannot completely discount the possibility that the fish used were more adapted to either salmon- or trout-mussels. We recorded host species–dependent mortality during the parasitic phase of the glochidia. We also recorded the total number of juveniles that excysted from Atlantic salmon and brown trout. Using parentage analysis, we examined whether individual mothers infested both hosts and whether infestation was biased towards one or the other host species.

## Materials and methods

Host infestation experiments were performed at the FPM rearing station at Austevoll, Norway. Lake Kvernavatnet provides the water supply for the rearing station, and the water has a pH of 6.6 and alkalinity of 0.108 mmol/l, and the concentrations of aluminium, iron, calcium, magnesium and nitrate were as follows: Al 180 μg/l, Fe 200 μg/l, Ca 4.2 mg/l, Mg 1.8 mg/l, Na 12 mg/l and Nitrate-N 0.15 mg/l. All incoming water was UV-treated and filtered through a 30-μm mesh before use.

### Glochidial collection

Adult mussels were collected from the rivers Slørdalselva (*n* = 52; Orkland municipality, Trøndelag county) and Loneelva (*n* = 40, Osterøy municipality, Vestland county) in August 2015 and April 2014, respectively. Fertilisation of Slørdalselva FPM took place in the wild, whereas those from Loneelva were fertilised at the FPM rearing station. Both these FPM populations use Atlantic salmon as the principal host (Johnsen et al. 2008). The mussels were transferred to the FPM rearing station and placed in artificial rivers with flowing water. They were fed regularly with a diet containing Shellfish® 1800 (Reed Mariculture Inc., Campbell, CA, USA) and Nanno 3600 (Reed Mariculture Inc.). Gravid mussels started spatting in September 2015 at a mean water temperature of 15.7 °C. Glochidial strings were collected from spatting individuals and checked for maturation and viability (≥90%), using methods described by Watters and O’Dee (1999), before we used them to infest the host fish.

### Fish infestation

**Fig. 1:**
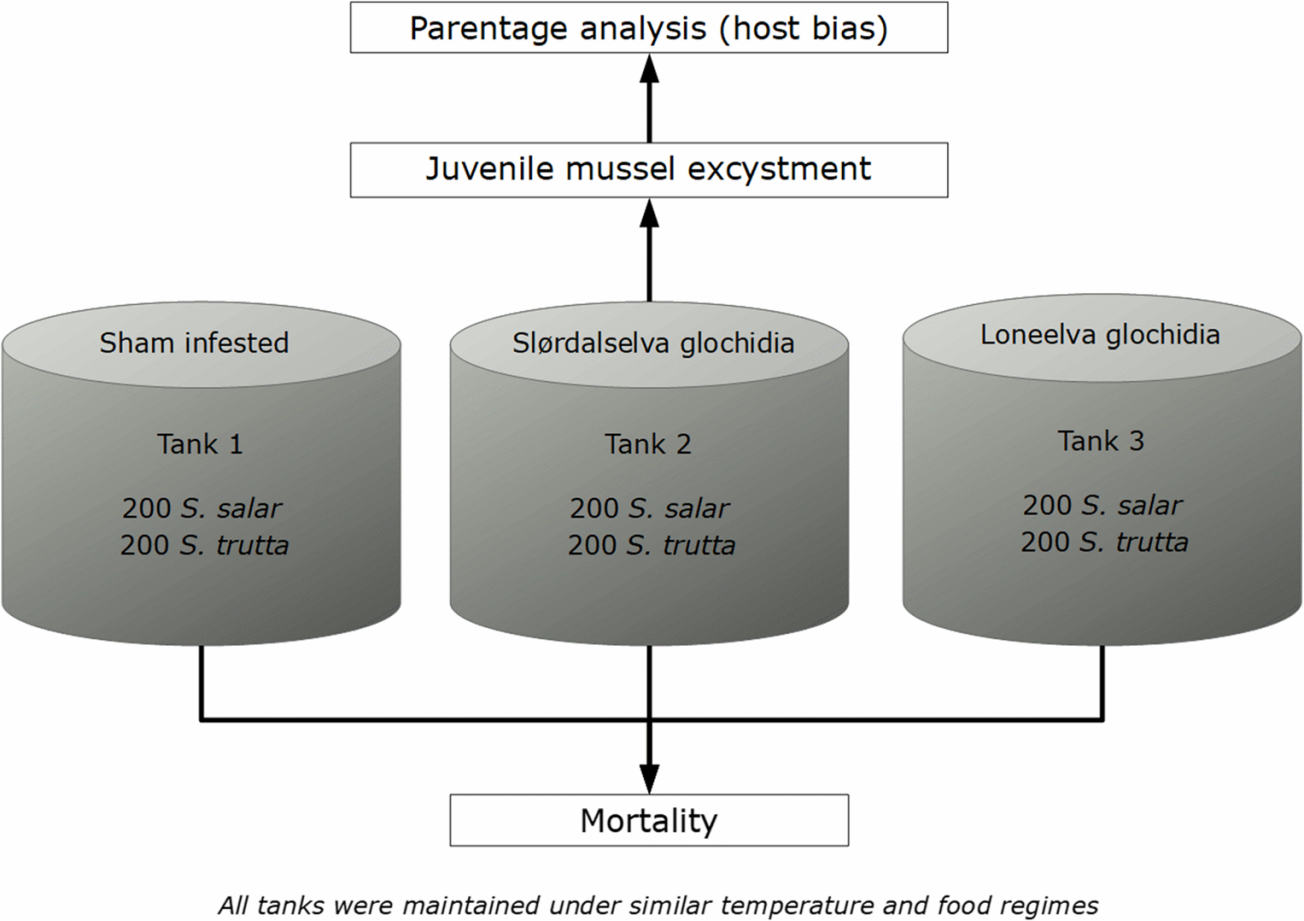
Schematic overview of the methods used in this experiment

Naïve hatchery-reared 0+ Atlantic salmon (Bjoreio, Vestland county, standard length 10.3 ± 7.1 cm) and brown trout (Botsvann, Agder county, standard length 11.3 ± 4.8 cm) from the Statkraft facility in Eidfjord municipality were transferred to the FPM rearing station in July 2015. The fish were kept in aerated tanks and fed until satiated. Before infesting the hosts with glochidia, 200 Atlantic salmon and 200 brown trout each, were transferred into three 4000 l (height, length, width = 1m × 2m × 2m) tanks: one as a control group and the other two as the infestation groups for the two FPM populations (Fig. 1). Infestation of the fish took place in September 2015. In order to infest the fish, we lowered the water levels in the tank and exposed the fish to glochidia (500 glochidia/l) for a period of 40 min with aeration (Taeubert et al. 2010). For the control groups, sham infestations were performed by exposing them to the same infestation conditions as the test groups, but without the presence of glochidia. All the groups (tests and control) were maintained under equal temperature and food conditions for the duration of the encystment period.

Post infestation, the fish were monitored daily, for number of mortalities, until the 18th of May 2016. Infestation status (presence/absence of glochidia) was recorded for a subsample of the fish that died (35–48% per host species and FPM population). The infestation status of the dead fish was examined by opening the gill flap and looking for the presence/absence of glochidia on all four gill arches. In addition, the gills were also examined under a dissecting microscope to confirm the infestation status. On the 18th of May 2016, the infestation status of all surviving fish exposed to Loneelva glochidia and of a random subsample of the fish exposed to Slørdalselva glochidia (44% of surviving trout and 87% of surviving salmon) was examined. Infestation status of the live fish, i.e. the presence/absence of glochidia, was checked only on the first two gill arches. Fish were first sedated with AQUI-S (2.5mg/l for 15 min), and then the gill flap was gently lifted to check for the presence/absence of glochidia. This was done visually, because it is easy to see encysted glochidia 9 months post infestation.

Since the live fish were checked very briefly for the presence of glochidia, it is likely that low levels of glochidial infestations were recorded as no infestation observed. Therefore, all fish exposed to glochidia were used to collect juvenile mussels. On 25 May 2016, fish exposed to glochidia were sorted and then transferred to separate juvenile mussel collecting chambers (90 l), i.e. one for trout and one for salmon. Fish were maintained in these tanks until the end of the excystment period, following the methodology originally described by Hruska (1999). Of the surviving fish, 76 out of 153 Atlantic salmon, and all the surviving (*n* = 100) brown trout exposed to glochidia from Slørdalselva were transferred into the mussel collecting chambers. The 200-μm collection sieves, under the collecting chambers, were inspected daily for the presence of excysted juvenile mussels, and mussels that excysted were collected and counted in order to get the total number of juvenile mussels that excysted from the salmon and trout harvesting chambers.

In order to calculate the infestation success of individual mothers on the two salmonid host species, we used fish infested with mussels from Slørdalselva. Juvenile mussels that excysted from Atlantic salmon and brown trout (*n* = 100 per host species) were collected in June and September 2016 for genetic analysis. Individual mussels were put into an Eppendorf tube containing 95% ethanol. Visceral swabs were taken for DNA collection from adult brood-mussels (parents) and stored in lysis buffer for later genetic analyses as explained by Karlsson et al. (2013). 

### Genetic analysis

Genetic analysis was done at the Norwegian Institute for Nature Research (NINA). DNA was extracted from the whole animal for juveniles and from cotton swabs for adults using DNeasy tissue kits (Qiagen). Mussels were genotyped at 15 microsatellite loci: MarMa3050, MarMa3621, MarMa4277, MarMa4322, MarMa2671, MarMa4143 and MarMa5280 (Geist et al. 2003) and Mm2201, Mm2230, Mm2235, Mm2240, Mm2207, Mm2210 and Mm2233, Mm2236 (Garlie 2010). PCR was carried out in two multiplexes (Karlsson et al. 2016). The following PCR protocol was used: 2-μl DNA, 4-μl Qiagen Multiplex Mastermix, 0.8-μl Primermix and 1.6-μl RNase-free water (Karlsson et al. 2016). The PCR was run on a Quattro Cycler (VWR) in the following conditions: denaturation for 15 min at 95 °C, followed by 30 cycles of 57 °C for 90 s and 72 °C for 60 s, and a final step of 60 °C for 30 min (Karlsson et al. 2016). For each multiplex, PCR products were visualised separately on an ABI 3130xl DNA analyser (Applied Biosystems) and sized using GENEMAPPER ver. 3.7 (Applied Biosystems).

### Assignment of offspring to mothers

Offspring were assigned to mothers using the likelihood-based approach in CERVUS 3.0 (Kalinowski et al. 2007). Adults were collected from the river after fertilisation had taken place. The collected adults were therefore known to include all mothers, while only an unknown fraction of fathers was collected. We first assigned parentage for offspring for which both the mother and the father had been sampled, using a parent pair analysis with unknown sex. We then performed a maternity analysis with complete sampling of maternal genotypes to assign the remaining offspring to mothers. All parentage analysis in CERVUS was performed with a mistyping rate of 0.01 and a critical Delta value for parentage assignment set for a confidence level of 95%. The sex of adults was inferred from assigned parentage. Adults that were assigned parentage in maternity analysis only, or in both maternity and parental pair analyses, were classified as females. Adults that were assigned parentage in parental pair analysis only were classified as males. Details of parentage analysis are presented in Wacker et al. (2018).

### Statistical analysis

We used the statistical package R, version 3.4.3 (R Core Team, 2017), for all statistical analysis. We used Fisher’s exact test to examine the probability of observing higher mortality in the exposed salmon and trout groups, when compared with control salmon and trout groups, respectively. This was performed separately for both Slørdalselva and Loneelva. We used a chi-square test to examine the null hypothesis that individual mothers infest both the host species with equal probability.

## Results

### Host mortality

**Fig. 2:**
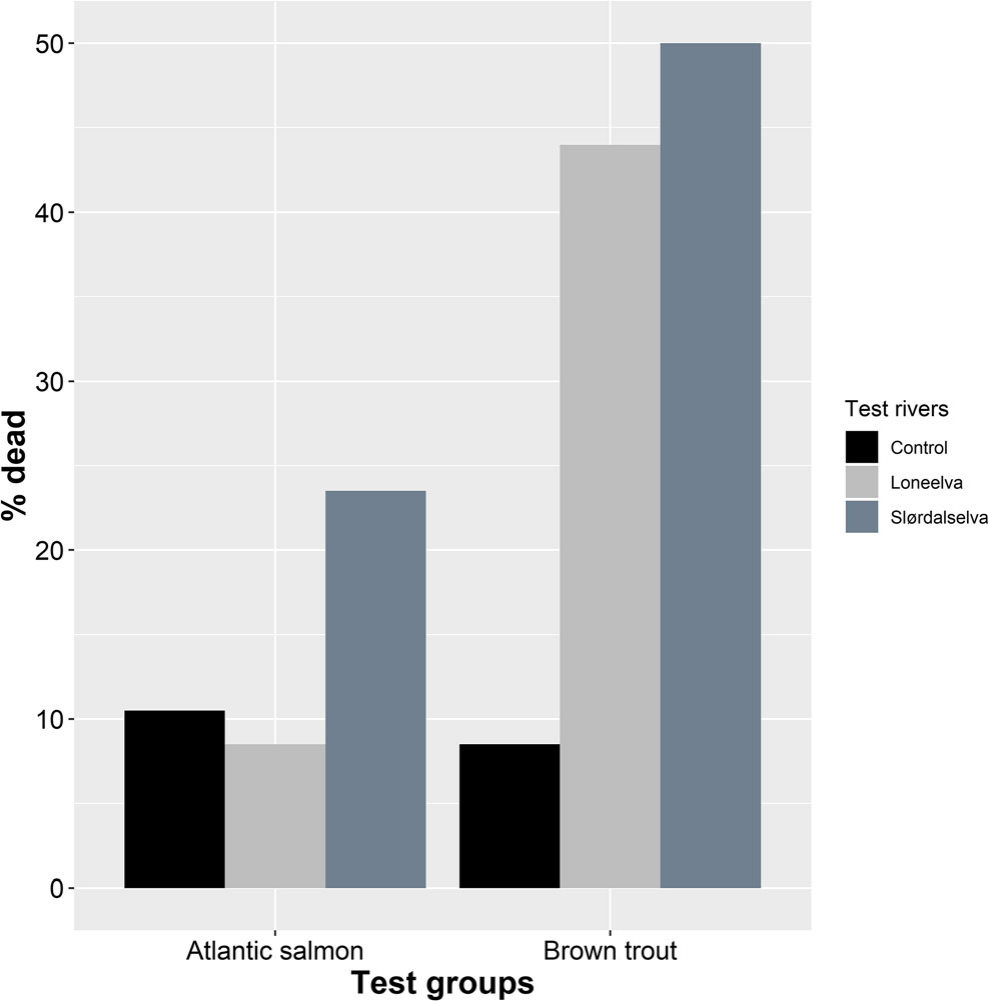
Bar plot showing the difference in host mortality (% dead) between the control Atlantic salmon and brown trout and those exposed to glochidia from the rivers Slørdalselva and Loneelva

Brown trout exposed to glochidia, from both the FPM populations, displayed a higher mortality compared to Atlantic salmon as well as control fish (Table 1). In Slørdalselva, the odds of an Atlantic salmon exposed to glochidia dying was 2.61 times that of a salmon in the control group (Fisher’s test: *p*-value = < 0.001, odds ratio = 2.61; Fig. 2). However, the odds of a brown trout exposed to glochidia dying was 10.69 times that of a trout dying in the control group (Fisher’s test: *p*-value < 0.001, odds ratio = 10.69; Fig. 2). In addition, the odds of a trout dying as a result of exposure to glochidia was 3.25 times that of a salmon dying when exposed to them (Fisher’s test: *p*-value < 0.001, odds ratio = 3.25). For Loneelva, we did not find any difference in the mortality of salmon exposed to glochidia and the control group (Fisher’s test: *p*-value = 0.6095, odds ratio = 0.7922, Fig. 2). However, the odds of a trout exposed to glochidia dying was 8.41 times that of a trout dying in the control group (Fisher’s test: *p*-value < 0.001, odds ratio = 8.41, Fig. 2). Similar to the previous result, the odds of a trout dying when exposed to glochidia was 8.41 times that of a salmon dying when exposed to them (Fisher’s test: *p*-value = < 0.001, odds ratio = 8.41).

**Table 1:** The total number of fish exposed to glochidia, total dead and total alive for the rivers Slørdalselva and Loneelva

Number of fish	Atlantic salmon			Brown trout		
	Control	Slørdalselva	Loneelva	Control	Slørdalselva	Loneelva
Total exposed	200	200	200	200	200	200
Total dead	21	47	17	17	100	88
Total alive	179	153	183	183	100	112

**Fig. 3:**
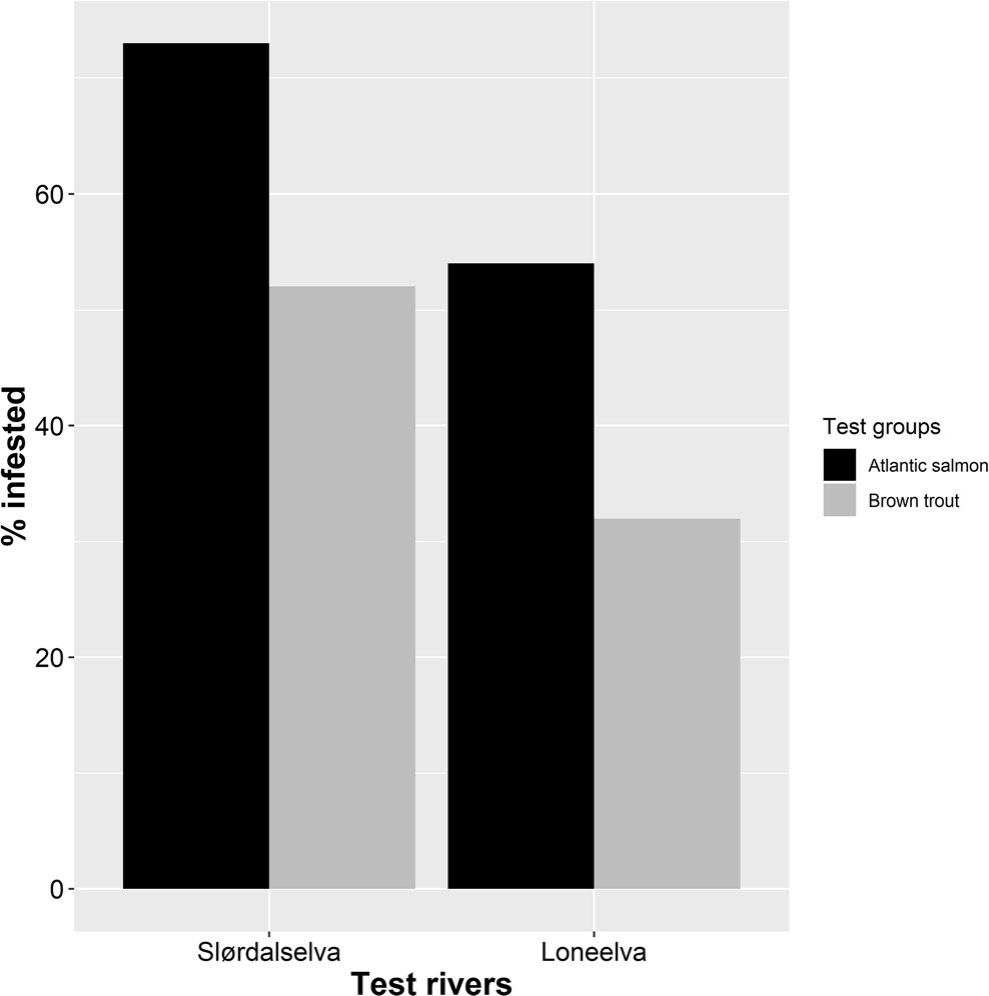
Bar plot showing the percentage of infested fish in the subsamples from Slørdalselva and Loneelva. In Slørdalselva, 73% of the salmon (109 out of 150 checked) and 54% of the brown trout (50 out of 92 checked) were infested. In Loneelva, 51% of the salmon (98 out of 189 checked) and 32% of the brown trout (48 out of 150 checked) were infested

**Fig. 4:**
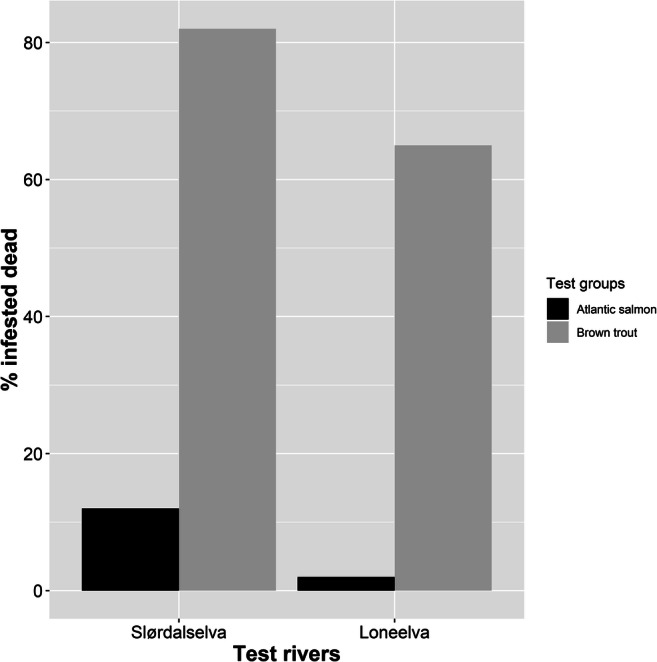
Bar plot of infested fish that died, shown as a percentage of the total number of infested fish. In Slørdalselva, 12% of the infested salmon (13 dead infested; 96 alive infested) and 82% of the infested trout (41 dead infested; 8 alive infested) died. In Loneelva, 2% of the infested salmon (2 dead infested; 98 alive infested) and 65% of the infested trout (31 dead infested; 48 alive infested) died

In our subsamples, a higher percentage of salmon were infested with glochidia compared to trout. In Slørdalselva, 73% of the salmon and 54% of the brown trout were infested, and in Loneelva 51% of the salmon and 32% of the trout were infested (Fig. 3). In Slørdalselva, the odds of a salmon becoming infested with glochidia, when exposed to them, was 2.23 times higher than a trout becoming infested (Fisher’s test: *p*-value = 0.005, odds ratio = 2.23). In Loneelva, the odds of a salmon becoming infested was 2.82 times that of a trout becoming infested (Fisher’s test: *p*-value = 0.0002, odds ratio = 2.82). In addition, when we examined only the infested fish in all the groups, we observed that a higher percentage of infested trout died compared to infested salmon. In Slørdalselva, 12% of the infested salmon versus 82% of the infested trout died. In Loneelva, 2% of the infested salmon versus 65% of the infested trout died (Fig. 4). In Slørdalselva, the odds of a trout dying as a result of glochidial infestation was 32.41 times that of a salmon dying due to it (Fisher’s test: *p*-value < 0.001, odds ratio = 32.41). In Loneelva, the odds of a brown trout dying as a result of infestation was 83.54 times that of a salmon dying as a result of it (Fisher’s test: *p*-value < 0.001, odds ratio = 83.54).

### Host bias

**Fig. 5:**
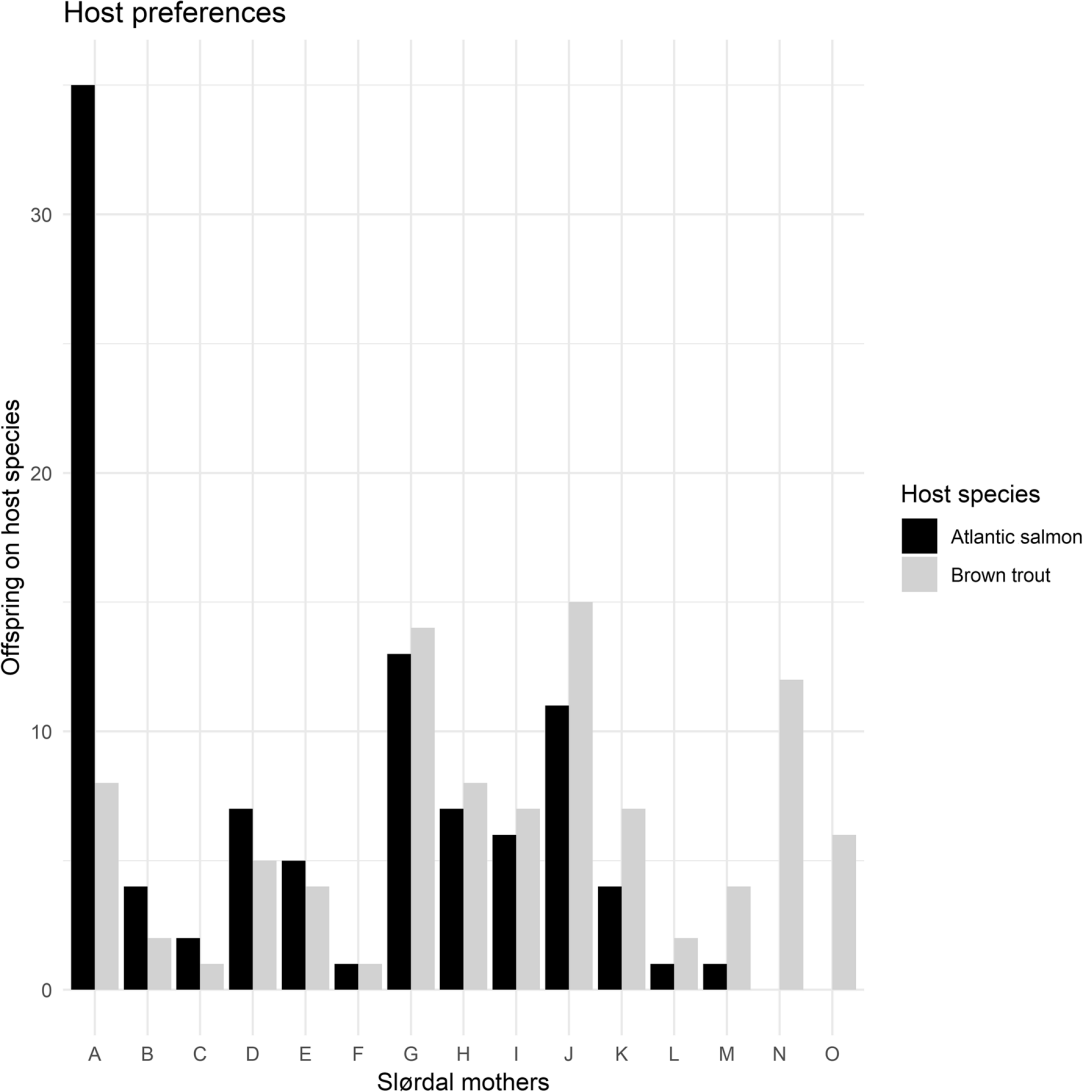
Bar plot showing distribution of number of offspring from individual mothers of FPM from the river Slørdalselva among 100 offspring that developed on Atlantic salmon (black bars) and 100 offspring developed on brown trout (grey bars)

The total number of juvenile Slørdalselva mussels collected from the harvesting chamber after the experiment was 23,780 for mussels that had developed on 76 Atlantic salmon and 14,909 for mussels that had developed on 100 brown trout. Consequently, the average number of juvenile mussels harvested per surviving fish was about two times higher for Atlantic salmon (313 juveniles/fish) compared to that of brown trout (149 juveniles/fish). Our results showed that individual mussel mothers from Slørdalselva infested both Atlantic salmon and brown trout. When 100 juvenile mussels collected from Atlantic salmon and 100 juvenile mussels collected from brown trout were assigned to individual mothers, mothers differed in the proportion of glochidia that infested Atlantic salmon and brown trout, respectively (*χ*2 = 40.141; df = 14, *p*-value = 0.0002, Fig. 3). For the majority of individual mothers, similar numbers of assigned offspring were collected from Atlantic salmon and from brown trout, but for some individuals, there was a strong bias (mothers A, M, N and O in Fig. 5).

## Discussion

The results of this study show that glochidial infestation resulted in host species–dependent mortality, in agreement with the hypothesis that virulence would be lower on the most suitable host. Brown trout exposed to and infested with glochidia displayed a significantly higher mortality compared to Atlantic salmon exposed to glochidia and the control groups. This was observed in both the FPM populations. In line with observations by Johnsen et al. (2008) and Eilertsen et al. (2018), Atlantic salmon was the most suitable host for Slørdalselva and Loneelva, and we observed that a higher proportion of salmon were infested compared to that of trout in both these rivers. In addition, Slørdalselva salmon also had the highest average number of excysted juvenile mussels per surviving host fish. Mussels primarily infested Atlantic salmon, but this bias varied between mothers, and some individual mothers infested brown trout to a higher degree compared to Atlantic salmon. Individual mussels may therefore produce offspring that can utilise both brown trout and Atlantic salmon, and not exclusively infest one or the other. This observation indicates that there is a large evolutionary potential for a shift in principal host where both hosts are being utilised to some extent.

The fitness of specialist parasites will often vary among different hosts depending on their suitability, and this can range from high fitness to zero fitness (Poulin 2007; Lefèvre et al. 2008; Schmid-Hempel 2011; Lievens et al. 2018). This has also been observed in several FPM studies, where glochidial fitness (quantitatively measured as abundance, prevalence and growth) was highest on the most suitable salmonid host species (Taeubert et al. 2010; Salonen et al. 2017; Taeubert and Geist 2017; Clements et al. 2018). Moreover, the results of this study have shown that glochidial virulence (host mortality) also varied among the two salmonid host species and was dependent on host suitability. However, the quantitative and qualitative (juvenile growth, lipid reserves) traits for measuring host suitability are not always correlated and can vary significantly between host species and between individuals of a suitable species (Taeubert et al. 2010; Douda 2015). For example, Douda (2015) observed that juveniles of *Unio crassus* and *Anodonta anatina* that developed on different host species, as well as individuals of a host species, varied in their lipid reserves as well as their early post-larval growth. In addition, they also observed that juveniles of *A. anatina* had the highest lipid levels on hosts with the lowest transformation success. Host species and individual hosts of a suitable species vary in terms of the conditions they provide for the developing glochidia. The variation in glochidial fitness is believed to be a result of host-parasite compatibility (Haag 2012), which in turn can depend on factors such as the genetic composition of the host and parasite, host factors (such as species, age, size, condition, infestation history, immune response, presence of other parasites), parasite factors (number of glochidia, virulence), environmental conditions (such as temperature) or a combination of these (Bauer and Vogel 1987; Combes 2000; Taeubert et al. 2010; Taeubert 2014; Marwaha et al. 2019). Nevertheless, our results show that the significantly higher mortalities, observed in the less suitable brown trout host compared to the principal Atlantic salmon host, were clearly a result of higher glochidial virulence on the less suitable host. These results are in line with Lievens et al. (2018), who observed that the microsporidian parasite *A. rigaudi* displayed higher virulence on the less suitable host *A. franciscana*, whereas on the suitable host (*A. parthenogenetica*) the parasitic virulence was moderate. They proposed that virulence could be related to the parasite’s degree of specialisation.

Virulence is not an exclusive trait of the parasite, but it is dependent on the parasite’s ability to inflict damage to the host, as well as the host’s ability to defend itself against the parasite (Schmid-Hempel 2011; Råberg and Stjernman 2012). Parasitic virulence not only has a negative influence on host fitness, but it can also result in low fitness of the parasite (Rutrecht and Brown 2009; Lievens et al. 2018). In this study, we saw that a higher glochidial virulence in the brown trout hosts not only led to higher mortality of infested fish but also resulted in glochidial mortality. Parasitic virulence can also be a contributing factor that influences host species composition in ecosystems, because differential virulence on hosts can result in parasite-mediated competition (Price et al. 1988; Schall 1992; Thomas et al. 1995; Lefèvre et al. 2008). The host species whose fitness is most affected by parasitic virulence is at a selective disadvantage compared to the less affected one (Price et al. 1988; Schall 1992; Thomas et al. 1995; Lefèvre et al. 2008). For example, Schall (1992) examined how the presence of the malaria parasite (*Plasmodium azurophilum*) affected the distribution of two highly competitive species of *Anolis* lizard species (*Anolis wattsi* and *Anolis gingivinus*). He observed that in the presence of *P. azurophilum* (which most commonly infects *A. gingivinus*), *A. wattsi* was present, and in the absence of the parasite, only *A. gingivinus* was present. This showed how parasite-mediated competition between species influenced their distribution. The results of this study suggest that salmon could possibly have a selective competitive advantage over trout in areas with dense salmon-mussels, due to the lower virulence and thus higher survival of salmon.

Generally in any host-parasite interaction, parasite traits (infectivity and virulence) and host traits (susceptibility and resistance) are governed by the interactions between the host and parasite genotypes and the interaction between their genotypes and the environment (Ewald 1983; Mackinnon et al. 2002; Day and Burns 2003; Perlman and Jaenike 2003; Lambrechts et al. 2006; Salvaudon et al. 2007). When there are no environmental influences, genotype × genotype interactions between the host and the parasite influence the phenotype of the host-parasite interaction, and this can vary across different host-parasite combinations; i.e. some parasitic genotypes will result in higher virulence on some hosts, and some host genotypes will be more susceptible to some parasitic infections (Engel and Wächtler 1989; Peever et al. 2000; Carius et al. 2001; Lambrechts et al. 2005; Salvaudon et al. 2005; Ebert 2008; Schmid-Hempel 2009; Taeubert et al. 2010; Schmid-Hempel 2011; Barribeau et al. 2014). Several authors have observed that unionid mussel species differ in their ability to infest different species/strains/populations of their fish hosts, and this was dependent on the interaction between a specific mussel population and the host fish species/strain/population (Engel and Wächtler 1989; Eckert 2003; Rogers et al. 2001; Taeubert et al. 2010; Douda et al. 2014, 2017; Schneider et al. 2017). For example, Engel and Wächtler (1989) examined the interaction between glochidia of different subspecies/strains of *Unio crassus* and their host fish *Leuciscus leuciscus* L. They observed that only the glochidia of *Unio crassus crassus* forma *maximus* were able to successfully metamorphose on this host, compared to *U. crassus crassus* which did not develop. Similar results have also been seen in other studies using *M. margaritifera* and *Salmo trutta* L. strains (Taeubert et al. 2010); *Unio crassus* and their hosts *Phoxinus phoxinus*, *Cottus gobio* (Schneider et al. 2017) and *Squalius cephalus* (Douda et al. 2014); *Sinanodonta woodiana* and hosts *Rhodeus ocellatus* (Douda et al. 2017), *Epioblasma florentina walkeri* and *Etheostoma flabellare* (Rogers et al. 2001); and *Cyprogenia aberti* and hosts *Percina phoxocephala*, *Percina caprodes* and *Etheostoma radiosum* (Eckert 2003). Some further examples from other host-parasite interactions, also showing differences in host and parasite traits, are *Daphnia magna* clones and *Pasteuria ramosa* (Carius et al. 2001; Decaestecker et al. 2003; Little et al. 2006; Ebert 2008), *Biomphalaria glabrata* and *Schistosoma mansoni* (Webster and Woolhouse 1998), *Bombus terrestris* and *Crithidia bombi* (Imhoof and Schmid-Hempel 1998), and Atlantic salmon with *Caligus elongatus* (MacKinnon et al. 1995), *Aeromonas salmonicida*, *Vibrio salmonicida* and *Renibacterium salmoninarum* (Fevolden et al. 1993). The difference in glochidial virulence observed in our study suggests a presence of host-parasite genotype × genotype interactions, and we believe that the aforementioned studies support our proposal that host-parasite genotype–specific interactions are an important underlying reason that determines the degree of glochidial virulence on the different salmonid hosts.

Hosts are often infested with multiple parasitic genotypes of the same species, and the interaction between coinfecting parasitic genotypes also influences the degree of virulence (Taylor et al. 2005; Lagrue et al. 2011; Bose and Schulte 2014; Råberg 2014). This is because a host is a limited resource, and the presence of two or more parasitic genotypes could lead to competition for resources (Taylor et al. 2005; Lagrue et al. 2011; Råberg 2014; Klemme and Karvonen, 2019). A higher virulence as a result of coinfecting parasitic genotypes have been observed in several studies (Ebert and Mangin 1997; Davies et al. 2002; Klemme and Karvonen, 2019. Thus, the interaction between the coinfecting parasitic genotypes will govern the degree of the infectivity or virulence and will vary for different genotype × genotype combinations (Taylor et al. 2005; Lagrue et al. 2011; Bose and Schulte, 2014; Råberg, 2014; Klemme and Karvonen 2019). In our experiment, glochidia came from several mothers, which in turn were fertilised by several fathers (Wacker et al. 2018). It is therefore highly likely that both the salmonid host species were infested with glochidia with different genotypes, and this was an added factor to the host-parasite interaction outcome. We believe that host-parasite genotype–specific interactions are an important underlying mechanism that determines the degree of FPM glochidial virulence on the two different salmonid hosts and partly explain host specificity.

Host immune strategy, such as resistance or tolerance to parasitic infection, is another important factor that can influence the degree of parasitic virulence (Lambrechts et al. 2006; Schmid-Hempel 2011; Hall and Ebert 2012). In host-parasite studies, resistance is described as the ability to prevent or reduce a given parasite, and tolerance is the ability to limit the damage caused by a given parasite (Råberg et al. 2009; Best et al. 2014; Jackson et al. 2014; Råberg 2014; Klemme and Karvonen 2016; Kutzer and Armitage 2016; Adelman and Hawley 2017). Several studies have shown that host immune response to infection by the same parasite differs among different host species (Fustish and Millemann 1978; Ellis and Stapleton 1988; Thomas et al. 1995; Buchmann and Uldal 1997; Bailey et al. 2019). These differences have been observed among various salmonid species with bacterial diseases (Ellis and Stapleton 1988; Bailey et al. 2019; Saleh et al. 2019) as well as with parasites (Johnson and Albright, 1992; Buchmann and Uldal 1997; Fast et al. 2006) including *M. margaritifera* (Fustish and Millemann 1978). Fustish and Millemann (1978) examined the immune response in Chinook salmon and coho salmon to glochidial infestation. They observed that the more resistant host (coho salmon) displayed severe hyperplasia and sloughed off glochidia within 4.5 days. In comparison, the more susceptible host (Chinook salmon) only displayed slight gill hyperplasia. Severe gill hyperplasia results in a decrease in respiratory gill surface area, leading to impaired gas exchange for which salmonid host fish have no adaptation (Taeubert and Geist 2013; Strzyzewska et al. 2016). Furthermore, the results from a previous experiment (Marwaha et al. 2019) showed us that 0+ brown trout were resistant to glochidial infestation, and the ‘resistant’ immune response led to a lower Fulton’s condition factor. When a resistant host mounts a strong immune response, this can result in the host’s own tissue being damaged or lower general host fitness. Moreover, resistant hosts may pay an energetic cost for being resistant. The higher virulence (mortality) we observed on brown trout could be a result of the host being resistant to glochidial infestation, as opposed to a higher degree of tolerance observed in Atlantic salmon which displayed a lower mortality. Typically high glochidial densities are associated with host mortalities (Treasurer et al. 2006; Taeubert and Geist 2013). However, we did not record this data in our experiment and are therefore unable to comment on the glochidial density–related mortalities of host fish.

Host suitability studies have shown that FPM populations display significant differences in host preference (Salonen et al. 2017; Taeubert and Geist 2017; Clements et al. 2018). However, it has not been clear if glochidia from an individual mother are able to successfully infest the different salmonid host species. The results of this study clearly show that mixed infestation on a population level is not explained by some mothers solely infesting trout and others solely infesting salmon. In fact, individual mothers from an FPM population with Atlantic salmon as the principal host were able to infest both the salmonid host species, but not with an equal number of larvae. In this study, we have only examined the effect of different mothers and not that of different fathers. Therefore, our data cannot exclude the possibility that offspring from individual fathers or parent pairs may have only infested either trout or salmon. FPMs are sperm casters, and female mussels obtain sperm via their inhalant siphon. A single mother can be fertilised by multiple males in a single breeding event (Young and Williams 1984b; Wacker et al. 2018), and the mothers in our study were also fertilised by several different males (Wacker et al. 2018) which might explain the observed variation in host specificity among offspring from the same mother.

In accordance with the Red Queen hypothesis (Koskella and Lively 2006; Rabajante et al. 2016; Anzia and Rabajente 2018), the tested salmon-mussel populations were best adapted to the most common host species in their habitat. Salmon- and trout-mussel populations not only display extreme host specificity and differences in genetic diversity but also differ in their timing of glochidial release and growth rates (Larsen 2002; Karlsson et al. 2014). It has been proposed that salmon- and trout-mussel populations have evolved entirely separately after the ice age, in concert with the respective colonisation history of their preferred host (Machordom et al. 2003). An alternative view is that salmon- and trout-mussels have a common colonisation history, with the observed differentiation a result of local adaptation (Wacker et al. 2019).

The results of this study show a clear difference in host-dependent glochidial virulence, with higher virulence in the less suitable host. We believe that this is the first study to report species-dependent host mortality as a result of glochidial infestation. We propose that this was a result of the interactions between host-parasite genotypes and the host immune response. Our results indicate that glochidial infestation could possibly result in parasite-mediated competition, because salmon would clearly have a fitness advantage (higher survival) over trout in areas of dense salmon-mussel populations. This study further showed that individual mothers infested both salmonid host species. Further studies to examine if offspring from individual fathers or parent pairs only infest either trout or salmon would improve our understanding of host-parasite compatibility. The FPM and their salmonid hosts provide a good model to study co-evolutionary interactions in a long-lived specialist parasite, which has a generation time that is almost 30 years longer than its host (Geist and Kuehn 2008). The results of this study highlight the importance of choosing the most suitable host when developing strategies for conserving endangered FPM populations and their host fish in the wild, as well as in captive breeding programmes. A further study that examines the differences in glochidial genotypes that infest salmon and trout, and their relation to virulence, would improve our understanding of genotype × genotype interactions and their influence on glochidial infectivity, virulence and host fitness.
